# COVID-19 Vaccine Hesitancy and Associated Factors among People with HIV in the United States: Findings from a National Survey

**DOI:** 10.3390/vaccines10030424

**Published:** 2022-03-10

**Authors:** Roman Shrestha, Jaimie P. Meyer, Sheela Shenoi, Antoine Khati, Frederick L. Altice, Colleen Mistler, Lydia Aoun-Barakat, Michael Virata, Miriam Olivares, Jeffrey A. Wickersham

**Affiliations:** 1 Department of Allied Health Sciences, University of Connecticut, Storrs, CT 06269, USA; antoine.khati@uconn.edu (A.K.); colleen.mistler@uconn.edu (C.M.); 2 Department of Internal Medicine, Yale School of Medicine, New Haven, CT 06510, USA; jaimie.meyer@yale.edu (J.P.M.); sheela.shenoi@yale.edu (S.S.); frederick.altice@yale.edu (F.L.A.); lydia.barakat@yale.edu (L.A.-B.); michael.virata@yale.edu (M.V.); jeffrey.wickersham@yale.edu (J.A.W.); 3 Department of Chronic Disease Epidemiology, Yale School of Public Health, New Haven, CT 06520, USA; 4 Marx Science and Social Science Library, Yale University Library, Yale University, New Haven, CT 06511, USA; miriam.olivares@yale.edu

**Keywords:** COVID-19, vaccination, hesitancy, people with HIV, health disparities, the United States

## Abstract

Introduction: Scaling up vaccination against COVID-19 is central to controlling the COVID-19 epidemic in the United States. Several vaccines are now approved for the prevention of COVID-19, but public concerns over safety and efficacy have heightened distrust and vaccine hesitancy. This is particularly concerning among people with HIV (PWH) who may be vulnerable to more severe COVID-19 disease. Here, we aimed to identify and understand COVID-19 vaccine hesitancy in a sample of PWH in the U.S. Methods: We conducted a cross-sectional online survey among PWH in the U.S. between 6 December 2020 and 8 January 2021. Measures included demographics, participants’ HIV and health-related attributes, COVID-19 history and experiences, COVID-19 vaccine-related concerns, and standardized measures of attitudes towards COVID-19 vaccines. Multivariate linear regression was used to identify factors associated with vaccine hesitancy in this sample. Results: Among the 1030 respondents, most were male (89.7%), White (66.0%), and identified as gay or lesbian (84.5%). Participants’ mean time living with HIV was 17.0 years (standard deviation (SD) = 11.1). The mean score for vaccine hesitancy was 1.5 (SD = 0.5; range: 1–5); 935 participants (90.8%) had a score greater than 1.0, indicating most participants had some degree of vaccine hesitancy. The final multivariate linear regression showed that greater vaccine hesitancy was associated with being Black (b = 0.149, *p* = 0.005), single (b = 0.070, *p* = 0.018), politically conservative (b = 0.157, *p* = 0.010), “anti-vaxxer” (b = 1.791, *p* < 0.001), concern about side effects (b = 0.226, *p* < 0.001), concern about safety (b = 0.260, *p* < 0.001), and being worried that the vaccine will not be effective (b = 0.169, *p* = 0.008) and they were being experimented on (b = 0.287, *p* < 0.001). Participants who were male White (b = −0.093, *p* = 0.008) and university graduates (b = −0.093, *p* < 0.001) and had a CD4 count of 200 cells/mm^3^ (b = −0.082, *p* = 0.048) and a liberal political orientation (b = −0.131, *p* < 0.001) were associated with lower vaccine hesitancy. Conclusions: Our findings provide important insights regarding COVID-19 vaccine hesitancy among PWH. Further efforts are required to understand how various social, political, and psychological factors contribute to COVID-19 vaccine hesitancy among key populations.

## 1. Introduction

Almost two years after the initial outbreak of SARS-CoV-2, the virus that causes COVID-19, several variations of the virus that appear to be more infectious continue to pose a grave threat to global public health. The World Health Organization (WHO, Geneva, Switzerland) and the U.S. Food and Drug Administration (FDA, Silver Spring, MD, USA) have recommended effective vaccine coverage as the single most important strategy to control the ongoing COVID-19 pandemic. Several vaccines are now approved under the emergency authorization for administration to the public [1], and federal, state, and local governments have prioritized their effort to improve vaccination rates [2]. However, minority groups who choose not to be vaccinated or do not have access to COVID-19 vaccines may complicate those efforts [3]. Substantial barriers to vaccine uptake include one’s ability to access the vaccine (e.g., transportation, cost, and location of services). In addition to these concerns, a significant barrier to uptake, particularly among minority populations, is vaccine hesitancy, which refers to the delay in the acceptance or refusal of vaccines despite availability [4], at least partly attributed to mistrust in the health care system and in the vaccine itself.

The accelerated development and approval of COVID-19 vaccines has led to increased skepticism about the safety and effectiveness of these vaccines, particularly among people with lower socioeconomic and education status, minority racial and ethnic populations, and persons with co-morbidities [5,6,7,8]. Latkin et al. [6] used a social-ecological model to describe the lack of trust in the COVID-19 vaccine on individual (e.g., concerns about side effects and purity of vaccine ingredients, fear of vaccine-induced infection, preventive misconception, and medical and government mistrust), social (e.g., norms of social approval of vaccine), and societal (e.g., trust in sources of information and equitable access to the vaccine) levels [6]. Groups with higher levels of COVID-19 vaccine hesitancy are often the same groups with higher co-morbidities rates due to various issues related to the intersectional stigma, accessibility, and affordability of healthcare services [9,10,11,12,13].

Individuals with some medical co-morbidities are at greater risk of a severe disease once infected with COVID-19, particularly those with compromised immune systems [14,15,16,17]. People with HIV (PWH) may be at higher risk of developing a severe case of COVID-19 due to overlapping co-morbidities, especially among those with unsuppressed HIV [17,18,19,20]. Additionally, COVID-19 has also disrupted healthcare services for PWH, including behavioral therapy [19,21]. Nonetheless, barriers to vaccination (e.g., accessibility, affordability, and mistrust) among PWH are limiting vaccine uptake, increasing their risk for adverse consequences, and greater rates of COVID-19 deaths among this population [16,22,23]. These factors place this subgroup in a unique circumstance to maximize efforts to avoid compounded threats to their immune systems [18]. Additionally, ongoing systemic discrimination and a perceived failure of health care organizations to build trust with PWH contribute to medical mistrust and potential hesitancy to receive COVID-19 vaccines among PWH [8,23]. Understanding and addressing vaccine hesitancy is important to ensure improved vaccine uptake. Given the increased risk for compounded consequences of COVID-19 among PWH, we sought to investigate vaccine hesitancy among a national sample of PWH in the United States.

## 2. Methods

### 2.1. Study Design and Participants

We conducted a cross-sectional online survey of PWH living in the United States between December 2020 and January 2021 to evaluate their attitudes toward the COVID-19 vaccine. At the time of the study, the FDA was in the process of granting emergency use authorization to two mRNA vaccines (Pfizer/BioNTech: 11 December 2020 and Moderna: 18 December 2020). In the U.S., healthcare workers and nursing home residents were prioritized for initial vaccination.

### 2.2. Study Procedures

Study procedures are reported as per the Checklist for Reporting Results of Internet E-Surveys (CHERRIES) guidelines [24]. We used G*Power 3.1 (Heinrich Heine Universitat Dusseldorf, Düsseldorf, Germany) [25,26] to conduct a power analysis to determine the minimum sample necessary to detect significance, assuming α = 0.05 and power (calculated as: 1–β) = 0.80, with a conservative effect size of d = 0.10 [27], resulting in a minimum sample size of 779. We recruited a convenience sample of PWH using an online recruitment strategy. Specifically, participants were recruited through paid advertisements on social media and social networking platforms (e.g., Facebook, Instagram, and Grindr), HIV/AIDS service and community-based organizations within the U.S., and peer referrals. On the social networking site, we used targeted banner advertisements that appeared in two ways: (1) a static ad on the right-hand panel of the website; or (2) an ad that resembled a standard post in users’ social media feed. Interested users who clicked on advertisements were directed to an eligibility self-screening tool and a brief online consent form hosted by Qualtrics. The eligibility criteria included adults aged 18 and over, currently residing within the U.S. and its dependent areas, and living with HIV.

Eligible volunteers completed an online consent form acknowledging that they understood the study’s purpose, risks, and benefits before completing the survey. Participation in the survey was voluntary, and participants were not paid for completing the survey but instead were eligible for a lottery to win 1 of 5 $100 Amazon gift cards; no participation was necessary to enter the random drawing. Those who declined to participate were provided instructions on how they could enter the lottery. Participants who completed the survey and were interested in entering the lottery were redirected to a different website where they entered an email address that was not linked in any way to their data. On average, participants took 10 min to complete the anonymous online survey. The study protocol and the consent form were approved by the Yale University Institutional Review Board.

We followed a protocol based on published standards for removing potentially duplicate cases while erring on the side of keeping, rather than removing, data in cases where a determination could not be made [28]. In particular, we first identified potential duplicates based on age, sexual orientation, and ethnicity. All cases sharing those features in common were manually examined, focusing on responses to other questions such as education, relationship status, income, device and browser information, and the survey duration.

**Figure 1:**
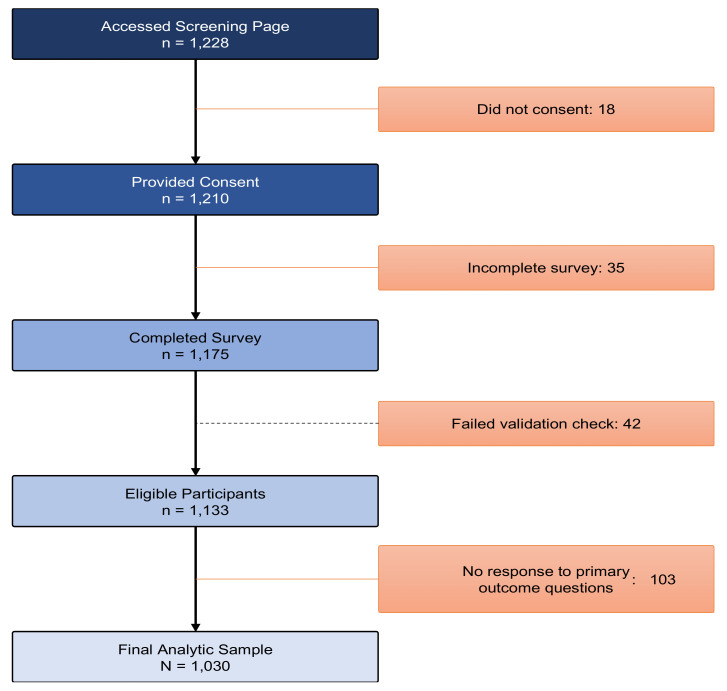
Flowchart of the participant recruitment (***N*** = 1030).

During the one-month recruitment period, 1228 participants entered the survey, and 1210 (98.5%) consented and completed the screening tool. Of the 1210 who completed screening and met inclusion criteria, 35 (2.8%) did not complete the survey. As such, the completion rate (i.e., the ratio of users who finished the survey/users who agreed to participate) of this survey was 97%. Of the 1175 who completed the survey, 42 participants were eliminated because they failed validation checks (e.g., survey duration), and 103 were not included in the analysis because they did not respond to the primary outcome question (vaccine-hesitancy items), leaving a final analytic sample of 1030 (Figure 1).

### 2.3. Study Measures

Sociodemographic and health characteristics: Participant characteristics included age, gender, race, sexual orientation, educational attainment, income, relationship status, political orientation (conservative, moderate, liberal, or other), years since HIV diagnosis, HIV viral load status (whether or not they are most recently virally suppressed), and whether they receive a vaccine for influenza annually. In addition to the recent (past 30 days) use of substance, participant alcohol use was measured using the AUDIT-C (screening cut-offs of ≥4 for men and of ≥3 for women correlated with the presence of an alcohol use disorder (AUD) [29]. Participants were also asked if they had been tested for COVID-19, if they had experienced any symptoms of COVID-19 since March 2020, and if they or anyone in their households had been diagnosed or died from COVID-19.

COVID-19 vaccine-related concerns were assessed using seven items with a dichotomized response of “Yes” and “No”, including “I am worried about side effects from the vaccine”, “I am against vaccines in general”, and “I don’t trust the government to distribute vaccine fairly”.

COVID-19 vaccine hesitancy was measured using a modified version of the 8-item Vaccine Confidence Scale (VCS) [30]. Originally designed to assess parental hesitancy toward early childhood vaccination, we modified the VCS to measure vaccine hesitancy in adult respondents. Three new items were added to this modified scale (e.g., “Vaccines are important for the health of others in my community”). Sample items from the modified VCS included “Vaccines are important for the health of others in my community”, “Vaccines are necessary to protect the health of individuals”, and “Vaccines are safe.” Responses were collected on a Likert-type scale ranging from 1 (strongly agree) to 5 (strongly disagree). To confirm the factor structure of the modified VCS, we conducted an exploratory factor analysis (EFA) using principal axis factoring and oblique rotation, which generated a 1-factor solution with a strong internal consistency (α = 0.873). The overall vaccine hesitancy score was obtained by generating a mean score for the 10 items, ranging from 1 (low vaccine hesitancy) to 5 (high vaccine hesitancy).

### 2.4. Data Analysis

We summarized the study participants’ characteristics with descriptive statistics such as the mean, standard deviations (SD), and frequencies and their bivariate correlations with COVID-19 vaccine hesitancy. Multivariate linear regression analysis was conducted to assess factors associated with the primary outcome—COVID-19 vaccine hesitancy, assessed as a continuous variable. Covariates for the multivariate model included (if *p* < 0.05 in a bivariate model) sociodemographic characteristics, political orientation, HIV and health-related attributes, personal experience with COVID-19, and concerns related to the COVID-19 vaccine. Estimates were evaluated for statistical significance based on probability criteria of *p* < 0.05. All analyses were performed using SPSS 25.0 (IBM, New York, NY, USA).

## 3. Results

**Figure 2:**
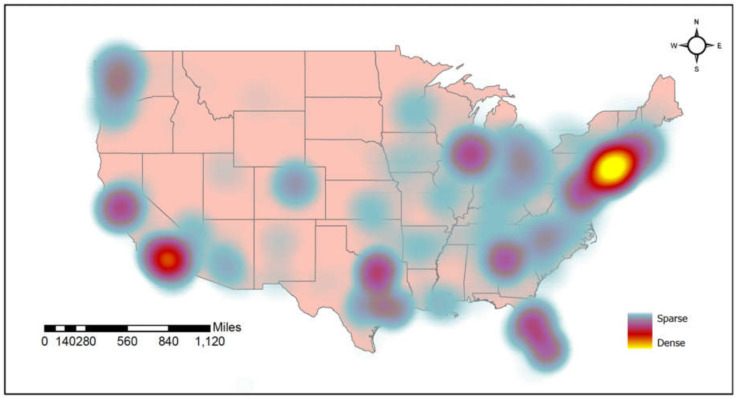
Spatial distribution of participant recruitment across the United States (***N*** = 1030).

Table 1 provides a summary of the characteristics of 1030 participants (median age = 53.0 y, interquartile range (IQR) = 60.0 − 41.0 y) recruited from across the United States (Figure 2). Participants had lived with HIV for 16.0 y (IQR = 26.0 − 7.0 y), and most were male (89.7%), White people (66.0%), and identified as gay or lesbian (84.5%). The majority of the participants self-reported having an undetectable HIV viral load (95.5%) and a CD4 count of 200 cells/mm^3^. Most (65.5%) had been previously tested for COVID-19, with 7.9% (81/1030) having tested positive at least once. Side effects (39.3%), safety (14.7%), and inequitable vaccine distribution by the government (13.6%) were the dominant concerns about COVID-19 vaccination.

The mean score of vaccine hesitancy was 1.5 (SD = 0.6; in the range of 1 to 5), although 935 participants (90.8%) had a score greater than 1.0, indicating most participants reported some degree of vaccine hesitancy. The univariate and multivariable linear regression showed that the greater COVID-19 vaccine hesitancy was associated with being Black (b = 0.149, *p* = 0.005), single (b = 0.070, *p* = 0.018), politically conservative (b = 0.157, *p* = 0.010), “anti-vaxxer” (b = 1.791, *p* < 0.001), concern about side-effects (b = 0.226, *p* < 0.001), concern about safety (b = 0.260, *p* < 0.001), and being worried that the vaccine will not be effective (b = 0.169, *p* = 0.008) and they were being experimented on (b = 0.287, *p* < 0.001). Participants who were male White (b = −0.093, *p* = 0.008) and university graduates (b = −0.093, *p* < 0.001) and had a CD4 count of 200 cells/mm^3^ (b = −0.082, *p* = 0.048) and a liberal political orientation (b = −0.131, *p* < 0.001) were associated with lower vaccine hesitancy (Table 2).

## 4. Discussion

Our study contributes to the limited literature on vaccine hesitancy among the nationwide sample of PWH living in the U.S., revealing most participants having some levels of hesitancy concerning COVID-19 vaccination. Previously reported vaccine hesitancy rates range from 22% to 42.4% among the adult American population [5,6,7,31] and 27.5% to 38.4% among PWH across various settings [32,33,34]. It is troubling that the vaccine hesitancy among PWH in our sample fell on the higher side of these ranges, as the pandemic adversely impacts HIV care with increasing loss to follow-up or disengagement [8,35]. Although most of the participants were virally suppressed, which indicates their active engagement in health care, they were hesitant about vaccination. The context of reduced vaccination rates due to hesitancy and increasingly disengaged PWH being at an increased risk of contracting COVID-19 [36] constitutes fertile grounds to exacerbate health-related inequalities in this population. Vaccination programs need to be culturally congruent and informed by people with lived experiences to reach people who would otherwise be hesitant.

Our results also indicated that race is associated with vaccine hesitancy, showing that Black PWH were more vaccine-hesitant compared to White PWH. Medical mistrust surrounding COVID-19 is high among Black PWH and presents an important and legitimate barrier to vaccination; it is rooted in systemic racism and arises as a sustained historical response to poverty, residential segregation, and previous and ongoing events featuring police violence [8]. Other factors may also contribute to this overall vaccine hesitancy, such as pre-existing reluctance towards vaccination due to prior side effects, such as decreased access to healthcare and less awareness and education regarding vaccine importance [5]. This association between race and vaccine hesitancy has been reported in the literature [5,6,7,31,37] and is concerning, as racial minorities (including Black PWH) have worse health outcomes associated with COVID-19 infection [5], with higher incidence and mortality rates among non-Latinx people who are Black, compared to among White people [38,39,40]. COVID-19 vaccine implementation should, therefore, imperatively consider racial differences in vaccine acceptance [6], especially in the context of chronic comorbidity such as HIV.

Participants who had higher education levels (i.e., bachelor’s degree or higher) reported lower levels of hesitancy in our sample. Other studies have found the same correlation [5,31,37], with other interlinked factors being lower income and rurality, which were not explored in our study. Decreased awareness regarding vaccination, reduced trust in and interaction with healthcare, and possible cost-related barriers within healthcare could explain why participants with a lower education level were more hesitant to get vaccinated [41,42,43,44,45]. Individuals in our sample who had an undetectable viral load or a CD4 count of >200 cells/mm^3^ were less hesitant to get the vaccine. This finding is concordant with prior reports in the literature (particularly regarding an undetectable viral load), validating that individuals who are more proactive about their HIV health are more likely to have higher intentions to be vaccinated [46].

The political ideology in our sample of PWH in the U.S. was associated with vaccine hesitancy. This finding is not surprising in the context of highly polarized sociopolitical grounds across states, differential risk perceptions among individuals, and the politicization of the COVID-19 pandemic. We found that self-defined political conservatism was associated with greater vaccine hesitancy, whereas liberalism was associated with less hesitancy. Individuals identified as politically conservative may have greater distrust in the government [47,48,49], perceive lower risks of COVID-19 infection and may be less likely to engage in preventative health behaviors [50], all of which might contribute to increased hesitancy in this subgroup. Interestingly, political ideology also appears to be a stronger predictor of vaccine acceptance than political party affiliation, as found by a recently published study, highlighting the complexity of the factors at play [6].

Participants who were not in a relationship at the time of our survey (i.e., single participants) were more hesitant to get the vaccine. We speculate that a substantial proportion of single people tend to live alone, which may render them less preoccupied with transmission to family, making vaccination less of a priority. In contrast, prior published research has reported that having children at home or being a parent are negative predictors for COVID-19 vaccination, potentially because of concern for vaccine side effects that might hinder their ability to care for their children [5,51]. Further research is needed to elucidate why being a parent or caregiver contributed to vaccine hesitancy among PWH. Vaccination efforts targeting whole family involvement could be considered in this case, perhaps through school campaigns, especially as the vaccine is approved for children aged 12 years and older [52].

General concerns about COVID-19 vaccine safety and their influence on vaccine hesitancy have been a frequent finding in the literature [6,7,31] and could be linked to the expedited vaccine development and rollout in response to the global health emergency [6]. In this study, several COVID-19 vaccine-related concerns were, in the same way, correlated with vaccine hesitancy. These included concerns about the efficacy, safety, and side effects of the vaccine. Although COVID-19 vaccine hesitancy among PWH across the globe is still advancing, multiple studies have already highlighted similar findings across various settings [32,33,34]. This finding becomes especially relevant in the context of the reported mistrust in COVID-19 or COVID-19 vaccine information sources, which has also been correlated with vaccine hesitancy [8,32].

Public doubts on vaccine safety have also been strongly linked to the use of social media in organizing offline action [53]. Being against vaccination in general and believing in conspiracy theories around COVID-19 (i.e., the belief of being a part of vaccine-related experiments) were also linked to increased hesitancy in participants. Further research is still needed to effectively reduce social media misinformation [54]. However, vaccine concerns and misinformation are addressable, as future vaccine programs or interventions are implemented [7].

This study was not without limitations. It was conducted, before COVID-19 vaccines were made widely available to the public. These limitations are inherent to a survey study design, such as self-reported information from participants, social desirability and recall biases, and the lack of a control group. Given the continuous nature of the primary outcome variable (i.e., vaccine hesitancy), we were unable to make a comparison between those who were vaccine-hesitant vs. non-hesitant. Furthermore, additional, unmeasured confounding factors could have influenced vaccine hesitancy on a personal level, such as previous experiences with vaccination and hesitancy presented against flu vaccine (but not other vaccines). Our sample was also a convenient sample of participants recruited using social media advertising. Therefore, this sample is limited to individuals with access to communication technologies (e.g., phones, tablets, and laptops) and the Internet. Finally, this survey likely excluded non-English speakers, as well as those who are illiterate or not able to read and understand its contents.

The findings from this study have important implications for the future implementation and planning of COVID-19 vaccine programs across the U.S. We were able to leverage an understanding of why PWH in the U.S. might be hesitant to receive vaccination and therefore attempt to guide future vaccination programs. A number of key predictors of vaccine hesitancy were identified and should be addressed in future efforts to mitigate the spread of COVID-19 through vaccination.

Efforts to raise awareness about the COVID-19 disease and vaccines through messaging and education should be tailored for high-risk groups, sexual minorities, and communities of color [37,55]. Culturally competent strategies that were shown to improve health outcomes and preventive behavior in minorities should be considered in the context of COVID-19 vaccines rollout as well [41,42,43,45]. Medical mistrust surrounding COVID-19 and negatively impacts vaccination among Black PWH in the U.S. should also be addressed by dismantling its causes at the societal level (e.g., poverty, residential segregation). Systemic racism should also be addressed at the national level through leadership voices. Community-based engagement, informed by people with lived experiences, can also be used to effectively tackle COVID-19-related and -unrelated inequities [8].

In addition to tailoring public health messaging for race and ethnicity, successful mass COVID-19 vaccination also requires public health interventions to respond to safety and efficacy concerns, as well as be adapted according to political orientation, gender-based differences, and political ideologies [5]. Responding to these different factors will require a multifaceted approach incorporating clinicians, public health professionals, and authorities.

In terms of general recommendations, it has been shown that advice concerning COVID-19 vaccination stemming from health care professionals is more trusted by the general American population (as compared to information from social media, for example) [6,37], including Black PWH [8]. Investing in provider-led interventions, emphasizing motivational interviewing, may support vaccine implementation efforts [31]. The increased use of telemedicine during the pandemic remains to be explored to advance vaccine implementation [6]. Potential recommendations to mitigate the effect of the fear of vaccine adverse events and safety concerns on vaccine hesitancy stress the importance of accurate and easily accessible and understandable information, as well as balancing risk and benefit information, positively framing adverse side effects, and dismantling related misinformation [56]. In order to overcome an ever-growing social media COVID-19 infodemic filled with misinformation, social media platforms must be held accountable for dismantling anti-vaccination content. Foreign disinformation should also be addressed at its source, since information warfare and anti-vaccination propaganda can be extremely harmful and fatal around the globe [53].

## 5. Conclusions

Our findings provide important insights regarding COVID-19 vaccine hesitancy among PWH, which represents a significant barrier to successfully implementing the nationwide vaccination campaign. As new variants emerge, there is an urgent need to ensure that PWH are prioritized for COVID-19 vaccination. Ongoing efforts must ensure that PWH continue to have equitable access to vaccines and up-to-date vaccine information. The findings from this study can inform how to implement mass vaccination campaigns and reach PWH who would benefit from vaccination by leveraging an understanding of independent factors associated with vaccine hesitancy.

## Figures and Tables

**Table 1:** Participant characteristics (***N*** = 1030).

Variables	Entire Sample (*N* = 1030)	
	Frequency	%
Sociodemographic		
Male sex	924	89.7
Race: Black	116	11.3
Race: White	680	66.0
Age (years)	53.0 (60.0 − 41.0)	
Education: bachelor’s degree or higher	507	49.2
Annual income: ≤$19,999	252	24.5
Sexual orientation: gay	870	84.5
Relationship status: single	552	53.6
Political orientation		
Conservative	72	7.0
Liberal	679	65.9
HIV & health-related attributes		
Has undetectable HIV viral load	984	95.5
CD4 count: >200 cells/mm^3^	804	78.1
Time living with HIV (years)	16.0 (26.0 − 7.0)	
Received an annual vaccine for influenza	867	84.2
Alcohol use disorder	303	29.4
Active drug use	45	4.4
COVID-19 history and experiences		
Ever been tested for COVID-19	675	65.5
Ever tested positive for COVID-19	81	7.9
Family member tested positive for COVID-19	141	13.7
Family member died from COVID-19	216	21.0
COVID-19 vaccine-related concerns		
“I am worried about side effects from the vaccine”	405	39.3
“I am concerned the vaccine will not be safe”	151	14.7
“I don’t trust government to distribute vaccine fairly”	140	13.6
“I don’t want to be experimented on”	96	9.3
“I don’t think the vaccine will be effective”	55	5.3
“I am against vaccines in general”	8	0.8
“I don’t trust medical doctors”	7	0.7

**Table 2:** Univariate and multivariable linear regression correlates of COVID-19 vaccine hesitancy in PWH in the United States (***N*** = 1030).

Variables	Univariate			Multivariable		
	Beta	S.E.	*p*	Beta	S.E.	*p*
Sociodemographic						
Male sex	−0.320	0.057	<0.001	−0.121	0.063	0.053
Race: Black	0.443	0.054	<0.001	0.149	0.052	0.005
Race: White	−0.300	0.036	<0.001	−0.093	0.035	0.008
Age (years)	−0.006	0.001	<0.001	−0.000	0.002	0.922
Education: bachelor’s degree or higher	−0.246	0.034	<0.001	−0.093	0.031	0.002
Annual income: ≤$19,999	0.214	0.040	<0.001	−0.001	0.031	0.977
Sexual orientation: gay	−0.267	0.048	<0.001	0.005	0.053	0.919
Relationship status: single	0.140	0.035	<0.001	0.070	0.029	0.018
Political orientation						
Conservative	0.393	0.068	<0.001	0.157	0.061	0.010
Liberal	−0.325	0.036	<0.001	-0.131	0.033	<0.001
HIV & health-related attributes						
Has undetectable HIV viral load	−0.241	0.085	0.004	−0.086	0.073	0.240
CD4 count: >200 cells/mm^3^	−0.190	0.052	<0.001	−0.082	0.041	0.048
Time living with HIV (years)	−0.004	0.002	0.004	−0.001	0.002	0.388
Received an annual vaccine for influenza	0.003	0.004	0.395			
Alcohol use disorder	0.015	0.038	0.693			
Active drug use	0.071	0.086	0.406			
COVID-19 history and experiences						
Ever been tested for COVID-19	−0.033	0.037	0.376			
Ever tested positive for COVID-19	0.080	0.065	0.221			
Family member tested positive for COVID-19	0.099	0.051	0.052			
Family member died from COVID-19	0.054	0.043	0.213			
COVID-19 vaccine-related concerns						
“I am worried about side effects from the vaccine”	0.446	0.033	<0.001	0.226	0.032	<0.001
“I am concerned the vaccine will not be safe”	0.594	0.046	<0.001	0.260	0.045	<0.001
“I don’t trust government to distribute vaccine fairly”	0.315	0.050	<0.001	0.065	0.042	0.127
“I don’t want to be experimented on”	0.724	0.056	<0.001	0.287	0.055	<0.001
“I don’t think the vaccine will be effective”	0.524	0.076	<0.001	0.169	0.063	0.008
“I am against vaccines in general”	2.291	0.186	<0.001	1.791	0.183	<0.001
“I don’t trust medical doctors”	1.161	0.210	<0.001	0.038	0.184	0.838

S.E., standard error.

## Data Availability

The data that support the findings of this study are available from the authors upon reasonable request.
